# Development and myogenesis of the vermiform *Buddenbrockia* (Myxozoa) and implications for cnidarian body plan evolution

**DOI:** 10.1186/2041-9139-3-10

**Published:** 2012-05-17

**Authors:** Alexander Gruhl, Beth Okamura

**Affiliations:** 1 Department of Zoology, Natural History Museum, Cromwell Road, London, SW7 5BD, UK

**Keywords:** Cnidaria, Myxozoa, *Buddenbrockia*, Endoparasitism, Development, Body axes, Symmetry, Chirality, Musculature, Mesoderm

## Abstract

**Background:**

The enigmatic wormlike parasite *Buddenbrockia plumatellae* has recently been shown to belong to the Myxozoa, which are now supported as a clade within Cnidaria. Most myxozoans are morphologically extremely simplified, lacking major metazoan features such as epithelial tissue layers, gut, nervous system, body axes and gonads. This hinders comparisons to free-living cnidarians and thus an understanding of myxozoan evolution and identification of their cnidarian sister group. However, *B. plumatellae* is less simplified than other myxozoans and therefore is of specific significance for such evolutionary considerations.

**Methods:**

We analyse and describe the development of major body plan features in *Buddenbrockia* worms using a combination of histology, electron microscopy and confocal microscopy.

**Results:**

Early developmental stages develop a primary body axis that shows a polarity, which is manifested as a gradient of tissue development, enabling distinction between the two worm tips. This polarity is maintained in adult worms, which, in addition, often develop a pore at the distal tip. The musculature comprises tetraradially arranged longitudinal muscle blocks consisting of independent myocytes embedded in the extracellular matrix between inner and outer epithelial tissue layers. The muscle fibres are obliquely oriented and in fully grown worms consistently form an angle of 12° with respect to the longitudinal axis of the worm in each muscle block and hence confer chirality. Connecting cells form a link between each muscle block and constitute four rows of cells that run in single file along the length of the worm. These connecting cells are remnants of the inner epithelial tissue layer and are anchored to the extracellular matrix. They are likely to have a biomechanical function.

**Conclusions:**

The polarised primary body axis represents an ancient feature present in the last common ancestor of Cnidaria and Bilateria. The tetraradial arrangement of musculature is consistent with a medusozoan affinity for Myxozoa. However, the chiral pattern of muscle fibre orientation is apparently novel within Cnidaria and could thus be a specific adaptation. The presence of independent myocytes instead of Cnidaria-like epitheliomuscular cells can be interpreted as further support for the presence of mesoderm in cnidarians, or it may represent convergent evolution to a bilaterian condition.

## Background

*Buddenbrockia plumatellae* Schröder, 1910 is a small vermiform parasite up to 3 mm long that was first described to occur in the body cavities of the freshwater bryozoans *Plumatella repens* and *P. fungosa*[1]*.* Following its discovery, this animal was encountered infrequently, but its peculiar morphology led to much speculation about its phylogenetic affinities. Thus *B. plumatellae* has been suggested to be a mesozoan, a nematode and a platyhelminth ([1-3], see [4] for review). More recent ultrastructural investigations provided a breakthrough by demonstrating that *B. plumatellae* produces spores with polar capsules that are diagnostic of myxozoans [5]. Additional support was afforded by molecular data [6]. The Myxozoa, like *B. plumatellae*, have also been a problematic taxon and were long assigned to protists. However, morphological similarities of polar capsules to cnidarian nematocysts [7,8], along with occurrence of nematocyst-specific genes in myxozoans [9] as well as further molecular sequence data [10,11], now provide strong evidence that myxozoans (including *Buddenbrockia*) group within Cnidaria most likely as part of the medusozoan radiation.

Myxozoan life cycles involve alternation between aquatic invertebrates (annelids or freshwater bryozoans) as definitive hosts and vertebrates (typically fish) as intermediate hosts [12]. Transmission stages between the hosts are physiologically inactive spores which consist of up to approximately 20 cells of three different types: (1) one to several amoeboid infective sporoplasms, (2) capsulogenic cells harbouring the nematocyst-like polar capsules which mediate attachment to the host and (3) valve cells that enclose the sporoplasms and capsulogenic cells. The majority of the approximately 2,180 known myxozoan species belong to the Myxosporea, which utilise annelids as their invertebrate hosts [13]. Myxosporean vegetative stages are morphologically simple, represented by either multicellular cysts with little to no grade of tissue organisation or multinucleated amoeboid plasmodia [12,13].

In contrast to the Myxosporea, the Malacosporea utilise freshwater bryozoans as invertebrate hosts, have more complex trophic stages that develop epithelial tissues, and produce soft (uncuticularised) spores that are similar in the vertebrate and invertebrate phases. There are currently three described species: *Buddenbrockia plumatellaeB. allmani* and *Tetracapsuloides bryosalmonae*, all of which infect freshwater bryozoans as definitive hosts. Salmonids are intermediate fish hosts for *T. bryosalmonae*[14-16], and there is indication that *B. plumatellae* exploits cyprinids [17]. In *B. allmani* and *T. bryosalmonae*, the trophic stages within the bryozoan are saclike and exhibit an outer wall of epithelial cells joined by cell-cell junctions and underlain by a basal lamina [18]. Worms of *B. plumatellae* are even more complex, with an additional internal epithelium that encompasses a central fluid-filled cavity and which, later in development, contributes to spore formation [5,18]. Between these two epithelial layers and embedded in the extracellular matrix (ECM) are four longitudinal muscle blocks that enable the worm to undergo rhythmic sinusoidal or spiralling movements within the host body cavity [5].

It is now clear that myxozoans have undergone a considerable radiation in connection with the evolution of endoparasitism, involving high rates of sequence evolution [10] as well as alteration or complete loss of major body plan features. For instance, they lack a gut and have no apparent nervous system, gonads, gametes or body axes. Furthermore, the aforementioned differences between Myxosporea and Malacosporea and the inclusion of *B. plumatellae* within the Malacosporea suggest that morphological simplification has occurred to varying degrees. Thus, some myxozoan subtaxa are more primitive in retaining plesiomorphic cnidarian or metazoan traits.

The vermiform stage of *B. plumatellae* is particularly significant for reconstructing ancestral character states and character polarities in Myxozoa. Such analyses are indispensable for identifying the myxozoan sister group, comparisons with parasitic cnidarians such as *Polypodium hydriforme* and understanding the evolution of parasitism. An active worm also constitutes a novel body plan within the Cnidaria [4]. This raises questions about how a functional worm can evolve from a cnidarian toolkit and whether there are similarities to or convergences with bilaterian worms. In this regard, the musculature is of special interest because it is topologically mesodermal and may thus relate to the debate about diploblasty vs. triploblasty in Cnidaria [19-21].

Current understanding of the morphology and development of *B. plumatellae* in bryozoan hosts is based on interpretations of two-dimensional data generated by light microscopy [1,2] and extensive transmission electron microscopy [5,18,22-25]. This previous work has clearly shown that early unicellular stages occur within the ECM beneath the peritoneum of the bryozoan body wall [22-25], where they proliferate by mitosis [25]. Worm development progresses with the appearance of small groups of cells that are almost certainly a result of divisions initiated by a unicellular founder [22]. These groups of cells become permanently associated to form a saclike structure with an external layer of cells (the future epidermis) linked by cell junctions and surrounding a loose collection of inner cells. Fibrous material between the epidermal and inner cells represents the forerunner of the basal lamina [22]. The sac elongates via division of the epidermal cells while the inner cells divide and form junctional complexes. As growth continues, the inner cells differentiate into four longitudinal muscle primordia and an inner epithelial layer that surrounds a central cavity [22]. With further growth, the muscles differentiate and the cells of the inner epithelium disaggregate and, with the exception of those positioned between the muscle blocks, become free in the lumen [25]. These sporogonic luminal cells proliferate and eventually undergo a complex series of events to produce multicellular spores [23,25].

In the present study, we employed confocal laser scanning microscopy to produce a coherent, three-dimensional understanding of *B. plumatellae* morphology and development across the entire length of worms at different developmental stages and to better resolve the architecture and arrangement of the musculature. This allows us to provide the first comprehensive picture of the bizarre musculature that underlies the movements of this aberrant cnidarian worm, to visualise the area of attachment to the bryozoan host, and to elucidate body symmetries and axis polarities.

## Methods

Colonies of various freshwater bryozoans were collected from the following localities: *Fredericella sultana* from Schiedersee, Schieder-Schwalenberg, Germany; *Hyalinella punctata* from Cowan Lake, Ohio, USA; and *Plumatella* sp. from the River Aabach, Switzerland. Colonies were kept in containers filled with water from the collection sites for 1 or 2 days. Colonies were inspected with a stereomicroscope to identify those with *B. plumatellae* infections. Opaque colonies of *F. sultana* and *Plumatella* sp. required dissection to locate infections. *B. plumatellae* worms could readily be seen in transparent colonies of *H*. *punctata*, which could then be dissected to obtain worms and infected zooids. This material was immediately fixed as described below.

A note on taxonomy:18S rDNA data [26] suggest that the vermiform malacosporean parasites in *Fredericella sultana* represent an undescribed species that is more closely related to *B. allmani* than to *B. plumatellae*. We refer to this as *Buddenbrockia* sp. 1. In this study, we did not find significant morphological differences between the worm stages of *B. plumatellae* and *Buddenbrockia* sp. 1, especially in the earlier stages, which were represented by most of our *Buddenbrockia* sp. 1 material. Worms collected from *Hyalinella punctata* and *Plumatella* sp. were morphologically indistinguishable, and there is little indication for different species status [26], especially as the hosts are very closely related [27]. To avoid confusion, we refer to the wormlike stages of *Buddenbrockia* sp. 1 and *B. plumatellae* collectively as “*Buddenbrockia* worms” in this study.

For transmission electron microscopy and histology, specimens were fixed in 2.5% glutaraldehyde in 0.01 M PBS at 4° for 4 hours. Specimens were rinsed several times in PBS and stored in PBS containing 0.05% NaN_3_ at 4°C until further processing. OsO_4_ (1%) was applied as a secondary fixative for 30 minutes, and specimens were subsequently dehydrated in an ethanol series and embedded into Epon via acetone. Series of semithin (0.5 μm) and ultrathin (60 to 70 nm) sections were obtained with a Leica Ultracut S microtome (Leica Microsystems, Wetzlar, Germany) and diamond knives. Semithin sections were mounted on glass slides and stained with toluidine blue (1% toluidine, 1% Na_2_B_4_O_7_, 20% sucrose) for 1 minute at 60°C on a hotplate. Stained sections were examined and photographed using an Olympus BX61 compound microscope equipped with charge-coupled device (CCD) camera (Olympus, Southend-on-Sea, UK). Ultrathin sections were mounted on formvar-coated single-slot grids, stained automatically with uranyl acetate and lead citrate (NanoFilm TEM Stainer; Ted Pella, Inc, Redding, CA, USA) and examined using a Hitachi 7100 transmission electron microscope (Hitachi Ltd, Tokyo, Japan) at 100 kV with a mounted Gatan CCD camera (Gatan, Inc, Pleasanton, CA, USA). For fluorescence staining and confocal microscopy, specimens were fixed with 4% paraformaldehyde in 0.01 M PBS at room temperature for 2 to 12 hours, rinsed in PBS and stored in PBS containing 0.05% NaN_3_ at 4°C. All further steps were carried out at room temperature. Specimens were permeabilised with PBS containing 0.1% Triton X-100 for 2 hours. In this step, ribonuclease A was added to a final concentration of 0.1 mg/ml to eliminate RNAs for later nuclear staining with propidium iodide. After rinsing in PBS, specimens were incubated with Alexa Fluor 488-labelled phalloidin (A12379; Invitrogen, Carlsbad, CA, USA) at concentrations of 2 to 4 U/ml for 4 to 12 hours. Phalloidin was thoroughly washed out with PBS, and specimens were stained with 2.5 μg/ml propidium iodide for 20 to 30 minutes and washed in PBS again. To detect dividing cells, some specimens were labelled with anti-phospho-histone H3 antibodies (06-570; Upstate, Temecula, CA, USA) diluted 1:1,000 in PBS containing 0.1% Triton X-100 and 2.5% bovine serum albumin and secondary antibody Alexa Fluor 488 goat anti-rabbit immunoglobulin G (A11008; Invitrogen) at 1:200 dilution. Both primary and secondary antibodies were incubated overnight at room temperature with several wash steps in between and afterward. Specimens were brought into mounting medium (90% glycerol, 10% PBS + NaN_3_, 0.25% 1,4-diazabicyclo[2.2.2]octane) via a graded glycerol-PBS series and mounted on glass slides with coverslips. Confocal image stacks were taken on a Leica TCS SP confocal laser scanning microscope (Leica Microsystems). Image data were analyzed using the MBF ImageJ (McMaster University, Ontario, Canada), Fiji ImageJ (Max Planck Institute of Molecular Cell Biology, Dresden, Germany), v3d (Howard Hughes Medical Institute, Ashburn, VA, USA) and Voreen (University of Münster, Germany and Linköping University, Sweden) software packages.

## Results

The description of the worm development is broken down into four morphologically distinguishable stages. To avoid redundant information, sections describing these stages do not give comprehensive accounts but focus on differences from previous stages. The duration of the entire developmental period is difficult to assess because early parasite stages are not easily visible in living hosts. However, development to mature worms from initial infection can occur in less than 31 days. Thus, worms were observed to develop in a bryozoan host that was infected in the field sometime during a period of 2 weeks. The bryozoan was subsequently kept in a laboratory mesocosm at 20°C with worms appearing after 17 days, none being observed 6 days earlier [25].

### Early stages

**Figure 1:**
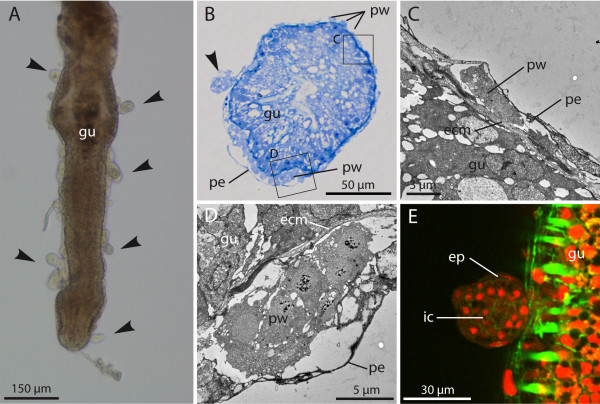
**Early developmental stages of*Buddenbrockia*sp. 1 in*Fredericella sultana.*(A)** Light micrograph showing spherical stages (arrowheads) attached to gut dissected from host. **(B)** Histological section of gut showing early preworm stages underneath peritoneum and spherical stage (arrowhead) that has penetrated through the peritoneum. **(C)** and **(D)** Transmission electron microscopy micrographs showing detail of the section shown in (B) as indicated by rectangles. Preworm stages reside underneath the peritoneum but on top of the basal lamina. **(E)** Confocal micrograph, optical section through early bilayered spherical stage attached to gut. F-actin (green); nuclei (red); ecm, extracellular matrix; ep, epidermis; gu, bryozoan gut; pe, bryozoan peritoneum; pw, preworm stage; ic, inner cells.

**Figure 2:**
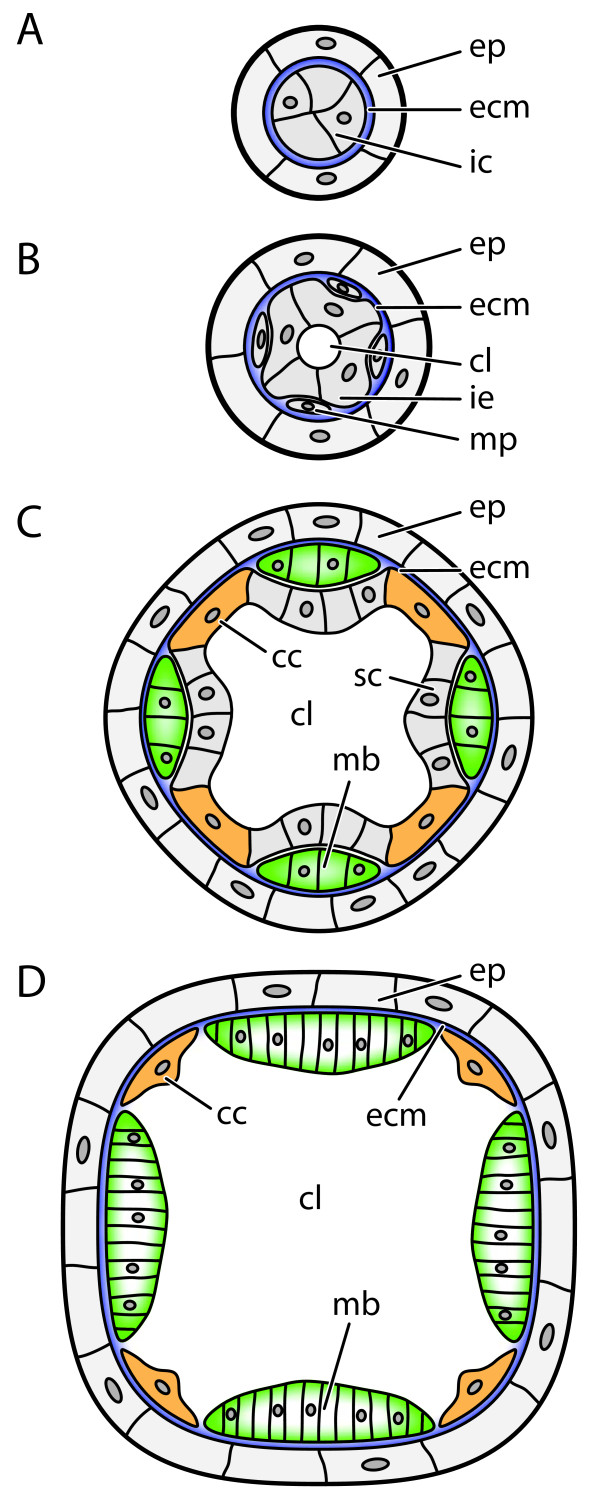
**Schematic representation of cross-sections summarizing tissue development in*Buddenbrockia*worms.(A)** Early stage with parenchymatous inner compartment. **(B)** Worm with inner epithelial tissue and muscle precursors. **(C)** Worm with musculature and inner epithelial tissue differentiated into connecting cells and sporogonic cells. **(D)** Worm with disintegrated inner tissue. cc, connecting cell; cl, central lumen; ecm, extracellular matrix; ep, epidermis; ie, inner epithelium; mb, muscle block; mp, muscle precursor; sc, sporogonic cell.

Early parasite stages clearly discernible by light microscopy are of spherical shape and attached to the host’s gut (Figures 1A, 1B and 1E) or body wall. They are 20 to 30 μm in diameter and appear bilayered with an outer epithelium-like cell layer and an inner cellular homogeneous (“mesenchymal”) compartment (Figure 1E). Serial histological sectioning, transmission electron microscopy and confocal microscopy of infected hosts reveal that although sometimes the parasites simply stick to the peritoneal surface, most of the attached stages are actually anchored between the peritoneum and the ECM layer (Figure 1B). In this compartment, even earlier uni- and multicellular preworm stages are found (Figures 1B to 1D). These very early stages are completely enclosed by the thin peritoneal layer of the host. The bilayered stages appear to develop once the growing cell mass penetrates the coelomic epithelium (Figures 1B and 2A). In all bryozoan host species examined, the attached stages were more often associated with the gut rather than with the body wall. If attached to the latter, these stages were mostly situated in the distal parts of the zooid, such as the tentacle sheath.

**Figure 3:**
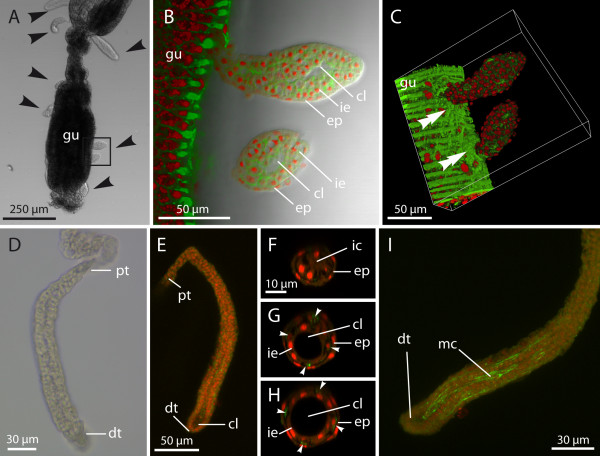
**Early developmental stages of*Buddenbrockia.*(A)** Light microscopy micrograph showing spherical as well as small, wormlike stages (*arrowheads*) attached to a gut dissected from *Fredericella sultana*. **(B)** Detail of **(A)** as indicated by rectangle. Confocal section merged with transmitted light microscopy micrograph showing details of tissue organisation. **(C)** Three-dimensional volume rendering of confocal image stack showing attachment to host tissue and host tissue reaction (double arrowheads). The tip of the worm is surrounded by slightly enlarged peritoneal cells that form cytoplasmic extensions with a higher actin content. **(D)** through **(I)** Early wormlike stages free-floating in body cavity of *Plumatella* sp. **(D)** Light micrograph. **(E)** through **(I)** Maximum-intensity projections of confocal microscopy image stacks showing tissue differentiation at the distal tip. **(F)** and **(G)** Confocal microscopy optical cross-sections through worm similar to the one shown in **(E)**. **(F)** Proximal end with compact mass of inner cells. **(G)** and **(H)** In the middle region and distal region, the inner cells have formed an epithelial layer encompassing a central lumen. Few cells (arrowheads), representing the muscle precursors, become situated in an intercalary position between the two epithelial layers. F-actin (green); nuclei (red); cl, central lumen; dt, distal tip; ep, epidermis; gu, bryozoan gut; ic inner cells; ie, inner epithelium; mc, myocyte; pt, proximal tip.

The parasites grow in length, acquiring a wormlike shape, and are found both attached (Figures 3A to 3C) and free-floating in the fluid-filled body cavity of the bryozoan host (Figures 3D to 3I). The percentage of free-floating worms in a host increases as these reach later stages of development; however, even nearly mature worms may still be attached. A considerable tissue reaction with cicatrised cells can be recognised in the peritoneum surrounding the proximal tip of attached worms (Figure 3C).

In the course of the elongation process, tissue development leads to a polarity in the worm body. At the distal, unattached end of the worm, the diameter increases slightly (Figures 3B, 2E-H and 2B) and the cells of the inner compartment differentiate. Most of these cells form an inner epithelial layer that encompasses a central lumen. However, a few cells do not become incorporated into this inner epithelium, but remain in an intercalary position between the inner epithelium and the epidermis and become surrounded by ECM (Figures 2B and 3F to 3H).

In later stages exceeding about 100 μm in length, phalloidin staining reveals the beginning of muscle fibre formation in those intercalary cells situated in the distal half of the worm (Figure 3I). The muscle fibres extend nearly to the distal tip of the worm, approximately where the inner cavity reaches its largest diameter. At the proximal end of the worm, the inner tissue is not differentiated into inner epithelium and muscle precursors, but remains a cell mass with a condensed “mesenchymal” appearance, as in the spherical stages. In these cells, enhanced cytoskeletal actin content underneath the cell membrane can be seen (Figure 3F).

### Worms with continuous inner epithelium

**Figure 4:**
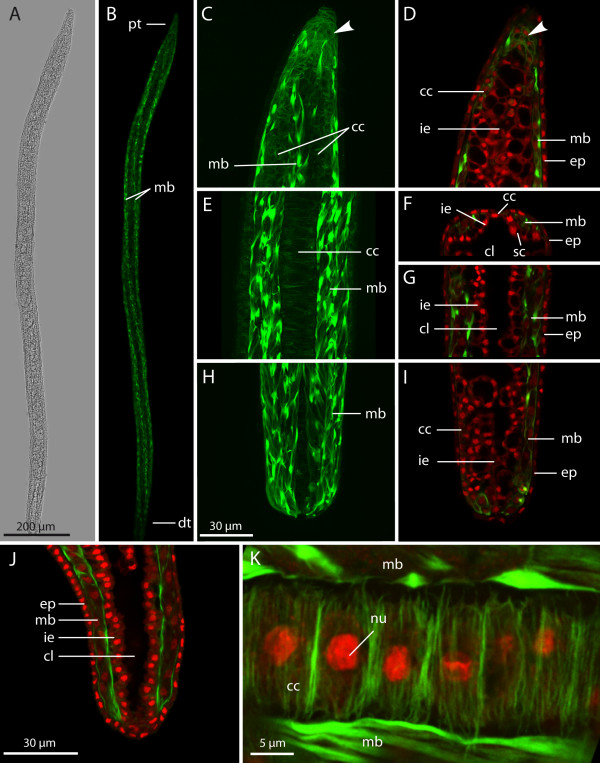
**Early stage*Buddenbrockia plumatellae*worm from*Plumatella*sp. (A)** Whole-mount light microscopy micrograph. **(B)** Confocal image of the same specimen, maximum-intensity projection showing body musculature. **(C)** through **(I)** Confocal images. **(C)**, **(E)** and **(H)** Maximum-intensity projections of whole three-dimensional image stacks. **(D)**, **(G)** and **(I)** Optical median horizontal sections. **(F)** Optical cross-section. Details of proximal tip **(C)** and **(D)** with mesenchymal inner cells (arrowheads), middle-body region **(E)**, **(F)** and **(G)** and distal tip **(H)** and **(I)**. Scale bar as in **(H)**. **(J)** Horizontal optical section through distal tip showing intact inner epithelium and nuclei in muscle cells. **(K)** Detail caption of row of connecting cells between neighbouring muscle blocks, horizontal aspect, maximum-intensity projection. F-actin (green); nuclei (red); cc, connecting cell; cl, central lumen; dt, distal tip; ep, epidermis; ie, inner epithelium; mb, muscle block; nu, nucleus; pt, proximal tip; sc, sporogonic cell.

Worms with a continuous inner epithelium measure 50 to 70 μm in diameter and up to 2 mm in length (Figure 4A). They are either attached or free-floating and already perform the characteristic spiralling movements inside the body cavity of the host, but less vigorously than fully mature worms. The basic histological organisation comprising an outer and an inner tissue layer is well-established throughout the entire length of the worm, except at the very tip of the proximal end (Figures 4C and 4D). A fluid-filled inner cavity is also present (Figures 4F and 4G). The musculature is clearly visible in phalloidin staining as four longitudinal blocks spanning the entire length of the worm (Figure 4B).

The muscle blocks are arranged circumferentially as four quadrants and are situated between epidermis and inner epithelium (Figures 2C and 4B, 4F, 4G and 4J). Each muscle block consists of two rows of obliquely oriented muscle cells. The cells are spindle-shaped and approximately 25 μm in length. Actin filaments are located underneath the cell membrane and are mainly concentrated at the apices or tips of the spindle, whereas the internal cytoplasm is devoid of F-actin. A nucleus is located centrally inside the cell (Figure 4J).

**Figure 5:**
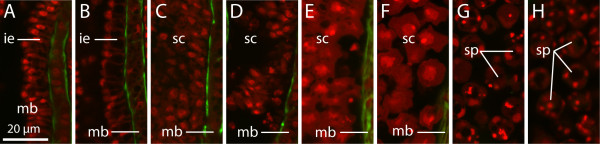
**Complete sequence of the development of the inner epithelium leading to spore formation in*Buddenbrockia plumatellae.*** Confocal optical horizontal sections. F-actin (green), nuclei (red). Initially, the inner epithelium is formed of a layer of cylindrical cells **(A)**, which then acquire large vacuoles on the basal side, restricting perinuclear cytoplasm to the apical side **(B)**. The cells detach from each other and float freely in the inner cavity **(C)** and **(D)**. They increase massively in size **(E)** and **(F)** and finally undergo cell division, leading to multicellular spores **(G)** and **(H)** in mature worms. ie, inner epithelium; mb, muscle block; sc, sporogonic cell; sp, spore.

The inner epithelium at first consists of uniformly cuboidal cells (Figures 4J and 5A). Later two different cell types can be distinguished. The cells that cover the muscle blocks toward the inner cavity of the worm are large and contain big vacuoles, which in confocal images appear nearly translucent in contrast to the cytoplasm (Figures 2C4D4F4G4I and 5B). In previous ultrastructural analyses, these cells have been referred to as type B [5,12]. Because of their function in later stages, we use the more descriptive term ‘sporogonic cells’. The nuclei as well as large parts of the cytoplasm are mostly situated in the apical region of the sporogonic cells. Those cells of the inner epithelium that reside between the neighbouring muscle blocks (Figures 2C4F and 4K), previously termed type A cells [5,12], differ in their cytological characteristics. We refer to them here as ‘connecting cells’. Their basal side is directly adjacent to the basal lamina underneath the epidermis, and they form a single cell row between the muscle blocks along almost the entire length of the worm. The connecting cells are smaller, lack the large vacuoles present in the sporogonic cells and exhibit an enhanced actin cytoskeleton directly underneath their cell membranes (Figure 4K) with fibres preferentially oriented at a right angle with respect to the longitudinal axis of the worm. Their stretched appearance suggests that these cells are firmly attached to the ECM surrounding the muscle blocks, a conclusion in keeping with the attachment to the basal lamina observed by Okamura *et al*. [5].

A comparison of both ends of the worm (Figures 4C, 4D, 4H and 4I) reveals the retention of a distinct body polarity in these stages. The distal tip (Figures 4H and 4I) appears rather blunt (rounded), with the internal cavity extending nearly to the end. The proximal end (Figures 4C and 4D) is more pointed, and the internal sporogonic cells do not reach the tip. In addition, owing to the decreasing diameter, the internal cavity is constricted and does not reach the tip. The inner compartment at the very tip appears as a “mesenchymal” cluster of cells similar to those in the very early spherical stages. Like the connecting cells, these cells show a much more enhanced actin cytoskeleton.

### Worms with disintegrated inner epithelium

**Figure 6:**
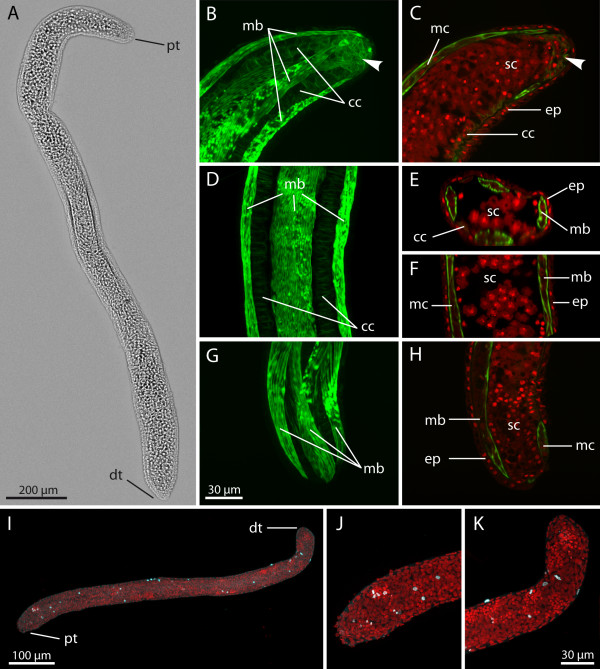
**Medium stage*Buddenbrockia*worms.(A)** through **(H)***Buddenbrockia plumatellae* from *Plumatella* sp. **(A)** Whole-mount light microscopic micrograph. **(B)** through **(H**) Confocal images. **(B)**, **(D)** and **(G)** Maximum-intensity projections of whole three-dimensional image stacks. **(C)**, **(F)** and **(H)** Optical median horizontal section. **(E)** Optical cross-section. Details of the proximal tip **(B and (C)**, middle-body region **(D)** through **(F)** and distal tip **(G)** and **(H)**. Scale bar is the same as in **(G)**. **(I)** through **(K)***Buddenbrockia* sp. from *Fredericella sultana.* Labelling with anti-phospho-histone H3 antibody (pH3) shows distribution of mitosing cells over the full length of the worm, with no apparent differences between the ends. **(I)** Overview. **(J)** Proximal tip. **(K)** Distal tip. F-actin (green); nuclei (red); pH3 immunoreactivity (cyan); cc, connecting cell; dt, distal tip; ep, epidermis; mb, muscle block; mc, myocyte; pt, proximal tip; sc, sporogonic cell.

More advanced worms differ mainly by their increased diameter (about 100 μm) and, in light microscopic images, by the more granular appearance of their inner contents, which is due to ongoing spore development (see below) (Figure 6A).

The muscle blocks have become wider and flatter in cross-section (Figure 6E). The muscle cells are still arranged obliquely and in two parallel rows (Figures 6B, 6D and 6G) but have elongated to about 50 μm and become much thinner. Cross-sections and longitudinal sections show F-actin to be located mainly underneath the cell surfaces facing the epidermis and central lumen, but not in regions facing neighbouring muscle cells (Figures 6C, 6E, 6F and 6H).

As also shown previously by ultrastructure [25], the inner epithelium has largely disintegrated and the sporogonic cells are now found floating in the central lumen, where they undergo spore formation (Figures 5C to 5F, and 6C6E6F and 6H). However, the connecting cells remain in place between the muscle blocks (Figures 2D6C to 6E), and their stretched appearance still demonstrates mechanical connection to the neighbouring muscle blocks (shown also by ultrastructure [5]). The sporogonic cells have enlarged nuclei and no longer appear vacuolated. There can be slight differences in the developmental state of these cells in different regions of the worm body.

The tips of the worm retain their appearance, with the proximal tip (Figures 6B and 6C) being more pointed than the distal one (Figures 6G and 6H) and exhibiting mesenchymal inner cells. To check for enhanced mitotic activity in these cells, immunolabelling with anti-phospho-histone H3 antibodies was conducted. This revealed that dividing cells are scattered along the entire length of the worms (Figure 6I) and that there are no apparent differences between proximal and distal tips (Figures 6J and 6K).

### Late-stage and mature worms

**Figure 7:**
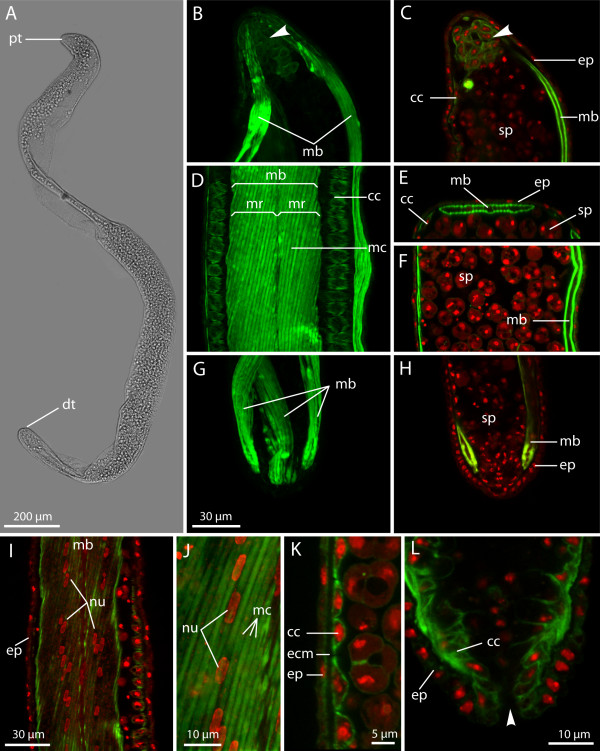
**Late-stage*Buddenbrockia plumatellae*worm from*Plumatella*sp. (A)** Whole mount, light micrograph. **(B)** through **(H)** Confocal images. **(B)**, **(D)** and **(G)** Maximum-intensity projections of whole three-dimensional image stacks. **(C)**, **(F)** and **(H)** Optical median horizontal section. **(E)** Optical cross section. Details of proximal tip **(B) and (C)**; middle-body region **(D)**, **(E)** and **(F)**; and distal tip **(G)** and **(H)**. Scale bar as in **(G)**. **(I)** Horizontal optical section through muscle block showing distribution of muscle cell nuclei. **(J)** Detail of **(I)**, maximum-intensity projection of muscle block. **(K)** Horizontal optical section through row of connecting cells. **(L)** Horizontal section through distal tip of mature worm showing porelike opening (arrowhead). F-actin (green); nuclei (red); cc, connecting cell; dt, distal tip; ecm, extracellular matrix; ep, epidermis; mb, muscle block; mc, myocyte; mr, myocyte row; nu, nucleus; pt, proximal tip; sp, spore.

Late-stage and mature worms (Figure 7) are characterised by the occurrence of multicellular spherical spores in the inner cavity, but they differ little in size to the previous stage. In many cases, the cavity is not filled uniformly with spores. Instead the spores may concentrate at one end or in certain regions of the worm body. Mature worms are nearly always free-swimming in the host body cavity and exhibit vigorous movements. They are able to leave the host through ruptures or the coelomic pores (see for example, Figure three in [28]) that are located at the anal side of the tentacle sheath and are normally used by the bryozoan to expel statoblasts.

**Figure 8:**
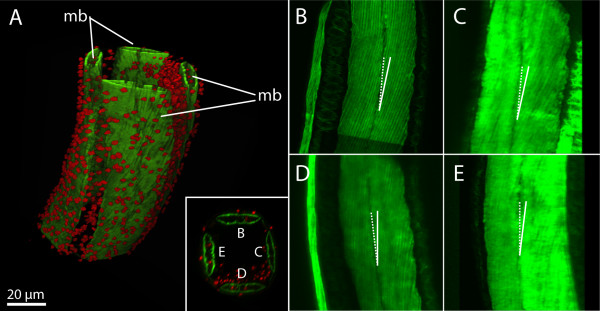
**Tetraradial symmetry pattern of muscle architecture in mature*Buddenbrockia plumatellae.*(A)** Three-dimensional volume rendering of confocal image stack from midsection of worm. **(B)** through **(E**) Maximum-intensity projections of image stack rotated in 90° steps to show arrangement and angle of muscle fibres in each muscle block as indicated in the inset in (A). F-actin (green), nuclei (red), mb, muscle block.

The muscle blocks are approximately 50 μm wide and about 5 μm thick and still consist of two rows of cells. The muscle cells are 2 μm wide, up to 130 μm long, and are oriented at an angle of 12° with respect to the longitudinal axis of the worm (Figure 7D). The nuclei have become oval in shape and are found in the middle of each muscle cell (Figures 7I and 7J). The distribution of myofilaments is, as in previous stages, underneath the outer and inner membranes of the muscle cell. The angle of the muscle cells is constant along the length of the worm and, as shown in Figure 8, similar in each of the four blocks, thus resulting in a tetraradially symmetric body organisation of the worm. In all worms of stages 2 to 4 examined (*n* = 32), the direction of the muscle cells is similar. In top view, the muscle cells always point from lower left to upper right (Figures 6D, 7D and 8). When seeing the musculature as an “interrupted” thread, this would correspond to a right-hand thread according to the terminology used for screws.

The connecting cells are still present and have not changed their appearance, apart from a slight increase in the amount of cytoskeletal F-actin (Figures 7D and 7K). The spores are now clearly multicellular (Figures 5G5H7C7E7F and 7H) with the typical malacosporean shape [18].

The polarity of the worms remains clearly visible in fluorescence-stained specimens even at this final stage (Figures 7B, 7C, 7G and 7H). In some worms, a porelike opening is found at the distal tip (Figure 7L).

## Discussion

### Attachment and early development

The present results regarding the early development of *Buddenbrockia* worms confirm most earlier observations made on the basis of light and electron microscopy [2,22-25]. The distribution of attached stages inside the host supports the view that the soft, exposed parts of the host body wall (for example, the tentacle sheath) and the gut are the main entry portal for the myxozoan spores, rather than the cystid wall, which is covered by a gelatinous or chitinous ectocyst. The presence of parasite stages (1) anchored in the ECM (2) attached to the surface of the coelomic epithelium and (3) free-floating in the body cavity suggests that very early and unicellular stages are able to migrate through the coelomic epithelium. Whether these develop into attached or free-floating stages may depend on their position within the host when growth commences. Earlier studies suggested that the onset of worm growth may be triggered by warmer temperature or availability of nutrients, since worms were observed to develop when subject to these conditions in a laboratory mesocosm [25]. There is strong evidence that such conditions trigger development of the closely related malacosporean *Tetracapsuloides bryosalmonae*[29-31].

The host tissue reaction may additionally strengthen the attachment of the worm, as it involves thickening and reinforcement of the cytoskeleton of the peritoneal cells that surround the attachment area. As worms of all lengths were found attached as well as free-floating, we suggest that detachment is not a scheduled event during development but occurs haphazardly, possibly facilitated by the increasingly vigorous movements of the worms. This hypothesis is supported by a higher proportion of detached older worms. The undifferentiated state and slightly ruptured appearance of the tissues at the proximal tip of detached worms renders it possible that a few cells could remain in the “wound” after detachment. Such material may therefore result in ongoing covert infection and the future growth of another worm. Note that covert infections are also likely to be achieved by the persistence of early stages, which we have observed in host tissues simultaneously with developing and mature worms. Such covert infections may promote long-term persistence in hosts such as occurs in *T. bryosalmonae*[31,32].

We have not found any evidence for the idea that the worms’ muscle cells could be derived from bryozoan muscle cells, as suggested by Morris and Adams [24]. The latter look rather different, and, in addition, muscle development occurs in the distal end of the worm. Furthermore, bryozoan nuclei are considerably larger than those of *Buddenbrockia* (see also [25] for ultrastructural differences), making potential chimeras easily detectable. We thus conclude that the musculature is a native *Buddenbrockia* feature that is likely to have been retained from a free-living cnidarian ancestor. In addition, our data do not support the idea that early multicellular stages form by the accumulation of migratory unicellular stages rather than by mitosis [24]. This would require the unicellular stages to move horizontally throughout the ECM, and a progression from loose clusters of cells to densely packed tissuelike stages would be expected. Such a progression is not evident in our data.

The present data demonstrate that the connecting cells persist in the mature worm and do not contribute to sporogony, as also shown by ultrastructure [5]. The possibility that they serve a mechanical function is supported by their attachment to the surrounding ECM, their stretched appearance and their pronounced actin cytoskeleton. However, the possibility of a neuronal function, as suggested by Schröder [2], cannot be ruled out completely.

### The presence of a polarised primary body axis

Our study shows that, during ontogeny, *Buddenbrockia* worms acquire a distinct polarity along their primary body axis, which is reflected by directional growth and a gradient of tissue differentiation. The inner cells as well as the musculature at the distal tip of developing worms are more differentiated than at the proximal tip. This polarity persists in later stages, and, in some fully mature worms, a porelike opening appears at the distal tip. However, from the present data, it cannot be determined whether this is a real pore or an opening as a result of rupture. Although internal cells at the distal pole sometimes differ in their morphology, we found no indication that these represent spermatids [2]. Anti-phospho-histone H3 staining does not give evidence of the presence of a distinct growth zone at either end of the worm. However, as the proportion of nuclei labelled with this method is low in comparison to methods such as bromodeoxyuridine labelling, which integrate over longer time periods, the latter might provide a more detailed picture in future studies.

Because *Buddenbrockia* lacks a gastrulalike stage, an intestinal tract and evidence of nervous structures, it is difficult to relate this axial polarity to that of other animals. Recent experimental and gene expression data (especially those based on Wnt/β-catenin signalling) demonstrate that the development of polyps from planulae and of asexual buds is in accord with early morphology-based hypotheses for a homology of the cnidarian oral-aboral axis and the bilaterian anteroposterior axis [33-36]. These data also suggest, although with lower confidence, that the cnidarian oral pole may correlate with the bilaterian posterior pole. Together with findings that growth at the posterior pole is most likely an ancestral character in bilaterian animals [37], this implies that the proximal tip of *Buddenbrockia* may correspond to the bilaterian posterior pole and thus the cnidarian oral pole. However, in cnidarians, no generalised pattern of either anterior or posterior growth has so far been demonstrated.

Another question is when and how polarity is determined. In cnidarians and bilaterians, the main body axis is either identical to or oriented at a particular angle to the embryonic animal-vegetal axis. This is achieved, for example, by gradients of maternally expressed transcription factors in the egg or the position of the egg with respect to the ovarial tissue (reviewed, for example, in [38,39]). However, the unicellular stages found in the bryozoan tissue are most likely derived from sporoplasms that came from spores produced in the vertebrate intermediate host [12]. They are amoeboid and do not show any recognisable polarity before mitotic divisions leading to early multicellular stages. It is therefore likely that polarisation is induced by external factors. This might be achieved via the orientation of the early stages in the bryozoan host tissue, such as with growth directed away from the basal lamina. Such a scenario could be tested by detailed comparison with closely related, saclike malacosporeans whose trophic stages lack a distinct body axis, such as the *Buddenbrockia* parasite of *Cristatella mucedo*[28]; *Buddenbrockia allmani* in *Lophopus crystallinus*[40]; or *Tetracapsuloides bryosalmonae*[18], which predominantly parasitises *Fredericella sultana*. For the latter species, data on early development indicate that presaccular stages are situated on the surface of the peritoneum rather than underneath as in *Buddenbrockia*[16], rendering the previous explanation possible.

### Mesodermal musculature and germ layers

As unequivocally indicated by the presence of nuclei in the muscle cells, the musculature of the *Buddenbrockia* worm is formed by independent myocytes and not by epitheliomuscular cells as in most other cnidarians [41,42]. If the epidermis is regarded as ectodermal and the inner epithelium (although functioning only as reproductive rather than digestive tissue) as endodermal, then the muscle cells are, at least by topological definition [43], mesodermal as they reside in the ECM between the two epithelial layers and are not connected to the latter. We note that the same argument applies if adaptation to parasitism has involved an inversion of ectoderm and endoderm as occurs in *Polypodium hydriforme*[44]. This may have bearing on questions concerning the evolution of metazoan body plans, since the diploblastic vs. triploblastic organisation of the last common ancestor of Cnidaria and Bilateria is highly debated (see, for example, [19-21,45]). Bilaterian mesoderm is usually characterised by (1) topology, (2) germ layer-specific derivation during gastrulation or (3) the presence of a common set of regulatory genes [45]. The commonly cited example for topological mesoderm in Cnidaria is the entocodon, a tissue that invaginates during hydrozoan medusa bud formation and gives rise to an independent striated subumbrellar musculature in the medusa (summarised in [20]). The musculature in free-living stages of the parasitic cnidarian *Polypodium hydriforme* also appears to be topologically mesodermal [46,47].

In contrast to the hydrozoan entocodon, the mesoderm of *Buddenbrockia* is formed early in ontogeny and might thus qualify as a bona fide germ layer potentially homologous to mesoderm in bilaterians. It also develops closer to the inner endodermal layer than to the outer epidermal layer. However, the lack of a distinct cleavage pattern, blastula-like stage and gastrulation-like process hinders further comparisons with cnidarian or bilaterian development. Of course, a mesodermal as opposed to a myoepithelial musculature may have functional advantages in a wormlike organism (see for example, [48]), thus promoting a convergent origin of this character in *Buddenbrockia* and bilaterians. The vermiform parasitic sea anemone *Edwardsiella lineata*, however, has typical anthozoan endodermal longitudinal muscles [49]. Gene expression data from *Buddenbrockia* should enable further insights into, for example, tissue homologies and axial patterning.

### Tetraradial symmetry and chirality

The arrangement of the muscle cells within the muscle blocks demonstrates that *Buddenbrockia* worms are characterised by tetraradial symmetry. Although traditionally regarded as radially symmetric, recent evidence, especially from developmental and gene expression studies, suggests that the ancestor of all cnidarians was a bilaterally symmetrical animal, a pattern still reflected in the organisation of many recent anthozoans [50-52] (but see [53]). Tetraradial symmetry must be regarded as having evolved in the Medusozoa, which form the sistergroup to Anthozoa [54]. The corroboration of a tetraradial symmetry in *Buddenbrockia* worms therefore provides further support for a medusozoan affinity of myxozoans [11].

A further interesting finding is the presence of a consistent handedness or chirality (mirror asymmetry) in the arrangement of the muscle fibres, which align in a right-handed thread in all individuals examined in this study. The coincidence of radial symmetry, or mathematically more precisely, rotational symmetry, together with mirror symmetry, may jointly characterise objects, with examples being a regular geometric star or the majority of radially symmetrical animals. However, the two types of symmetry are not necessarily linked. Many rotationally symmetrical objects are chiral and exhibit rotationally repeated elements with no mirror symmetry. Such chiral forms exist in two (dextral vs. sinistral) enantiomorphs (see [55] for a review of symmetry patterns).

Chirality is a well known phenomenon in many bilaterian animals (e.g. molluscs, annelids, pterobranchs, nematodes, vertebrates) [56], but has so far only rarely been described in non-bilaterians. An interesting non-bilaterian case includes certain conulariids, which are a fossil group inferred to have been scyphozoans. Thus, the tetraradially symmetrical skeleton of the conulariid *Metaconularia anomala* shows torsion along the longitudinal axis [57]. *M. anomala* also exhibits a strong preference for a sinistral coil [57]. Other nonbilaterian examples include the siphonophore *Bargmannia elongata*, in which bud formation leads to consistently asymmetrical colony forms [58], and fossils probably belonging to the ctenophoran lineage and which possess arms that coil preferentially dextrally [59].

Fixed chiralities such as the one described here in *Buddenbrockia* are in almost all cases heritable [60]. So, is it likely that chirality is adaptive? A similar angle of the muscle fibres throughout the body is probably strongly selected for because of its functional significance for spiralling movements. However, such movement should be achieved regardless of whether the angle is dextral or sinistral. A possible explanation for the dominance of one chiral form might be if concerted movements of multiple worms within a host minimise interference.

How is the chirality established in *Buddenbrockia* worms? In most bilaterians, where the developmental mechanism leading to chirality is known, symmetry breaking occurs after the establishment of the dorsoventral axis (see, for example, [61]). However, a deeper underlying mechanism that establishes chirality on a subcellular level is generally inferred. This has not been clearly identified and may differ from case to case. The main theories are that cellular chirality is a result of (1) molecular chirality of the cilium, (2) cytoskeletal asymmetries leading to a differential distribution of ion channels and/or pumps on one side of a blastomere or (3) nonidentical blastomeres produced by different epigenetically imprinted patterns [62]. The ciliary model can clearly be ruled out for *Buddenbrockia*, as cilia and centrosomes are lacking in all myxozoans [12]. As the chirality is reflected only in the arrangement of the muscle cells, it could be explained by a directional shift in the cleavage plane in mitoses leading to the muscle cell lineage.

## Conclusions

Our study has provided fundamental insights into the evolution of polarity, symmetry and tissue layers in the lower Metazoa. The presence of a polarised primary body axis represents a unique retention in Myxozoa of this ancient feature, present at least in the last common ancestor of Cnidaria and Bilateria if not in the Metazoa. In the absence of clear morphological features, however, clarification of the homologies of the proximal and distal tips with the cnidarian oral or aboral poles will require gene expression studies. The mechanism by which the primary axis is established is probably deviant from other metazoans and could provide insights into how this character was lost in all other myxozoans.

The tetraradial arrangement of musculature supports a medusozoan affinity for Myxozoa. The chiral pattern revealed in the orientation of muscle fibres has no known equivalents within Cnidaria, however, and could thus be an adaptation that allows the spiralling movements of the worm. The unique biomechanics of *Buddenbrockia* locomotion will be the subject of a forthcoming publication. Finally, the presence of independent myocytes instead of cnidarian-like epitheliomuscular cells can be interpreted as further support for the presence of mesoderm and thus triploblasty in cnidarians. An alternative explanation is that the topologically mesodermal muscle represents convergent evolution to a bilaterian bauplan.

## Abbreviations

PBS, phosphate-buffered saline..

## Competing interests

The authors declare no competing interests.

## Authors’ contributions

AG and BO have collected the animals. AG has conducted the microscopy. AG and BO have analysed the data and drafted the manuscript. All authors have read and approved the final manuscript.
